# Cast versus functional brace in the rehabilitation of patients treated non-operatively for a rupture of the Achilles tendon: protocol for the UK study of tendo achilles rehabilitation (UK STAR) multi-centre randomised trial

**DOI:** 10.1136/bmjopen-2017-019628

**Published:** 2017-10-24

**Authors:** Juul Achten, Nick R Parsons, Rebecca L Kearney, Michael Maia Schlüssel, Anna S Liew, Susan Dutton, Stavros Petrou, Benjamin Ollivere, Sarah E Lamb, Matthew L Costa

**Affiliations:** 1 Oxford Trauma, Nuffield Department of Orthopaedics, Rheumatology and Musculoskeletal Sciences, University of Oxford, Oxford, UK; 2 Statistics and Epidemiology Unit, Warwick Medical School, University of Warwick, Coventry, UK; 3 Warwick Clinical Trials Unit, Warwick Medical School, University of Warwick, Coventry, UK; 4 Oxford Clinical Trials Unit, Centre for Statistics in Medicine, Nuffield Department of Orthopaedics, Rheumatology and Musculoskeletal Sciences, University of Oxford, Oxford, UK; 5 Faculty of Medicine and Health Sciences, Queen’s Medical Centre, Nottingham, UK

**Keywords:** accident &emergency medicine, orthopaedic & trauma surgery

## Abstract

**Introduction:**

Achilles tendon rupture affects over 11 000 people yearly in the UK, and the incidence is increasing. Controversy remains with regard to the best rehabilitation strategy for these patients. In operatively treated patients, functional bracing provides better outcomes compared with plaster casts. However, the role of functional bracing in non-operatively managed patients is unclear. This is the protocol for a multicentre randomised trial of plaster cast immobilisation versus functional bracing for patients with a non-operatively managed Achilles tendon rupture.

**Methods and analysis:**

All adults presenting with a primary rupture of the Achilles tendon will be screened. Non-operatively treated patients will be eligible to take part in the trial. Broad eligibility criteria will ensure that the results of the study can be generalised to the wider patient population. Randomisation will be on a 1:1 basis. Both rehabilitation strategies are widely used within the National Health Service. Standardised protocols will be followed, and details of plaster material and brace will be as per the site’s usual practice.

A minimum of 330 patients will be randomised to obtain 90% power to detect a difference of 8 points in Achilles Tendon Total Rupture Score at 9 months. Quality of life and resource use will be collected at 3, 6 and 9 months. The differences between treatment groups will be assessed on an intention-to-treat basis. The results of the trial-based economic evaluation will be expressed in terms of incremental cost per quality-adjusted life-year gained.

**Ethics and dissemination:**

The National Research Ethic Committee approved this study on 18 March 2016 (16/SC/0109).

The National Institute for Health Research Health Technology Assessment monograph and a manuscript to a peer-reviewed journal will be submitted on completion of the trial (summer 2019). The results of this trial will substantially inform clinical practice on the clinical and cost-effectiveness of the treatment of this injury. This study has been registered on the International Standard Randomised Controlled Trial Number registry with reference no ISRCTN62639639.

**Strengths and limitations of this study**

- Broad eligibility criteria to ensure generalisability.
- Patient-centred outcomes in combination with complication data will be collected.
- Assessment of outcomes at multiple time points will allow for information on recovery profile.
- In addition to a comparison of clinical outcomes, a full cost evaluation will be performed.
- It will not be possible to blind patients to their allocated treatment, as the plaster cast/walking boot will be clearly visible.

## Background

The Achilles tendon is the largest tendon in the human body and transmits the powerful contractions of the calf muscles that are required for walking and running. When the tendon ruptures, it is painful and has an immediate and serious detrimental impact on daily activities of living.^1^ In the longer-term, tendon rupture results in prolonged periods off work and time away from sporting activity: average time away from work is between 4 and 8 weeks and time away from sport is between 26 and 39 weeks.^1^ This results in lost income and restricted daily activities in the early phase and reduced physical activity, with associated negative health and social consequences, in the long-term. For high-level sportsmen, it is frequently a ‘career-ending’ injury.

Achilles rupture affects over 11 000 people each year in the UK, and the incidence is increasing as the population remains more active into older age.^2^ It affects all age groups in a bimodal distribution; with the first peak in patients aged 30–40 years and the second 60–80 years.^2^ The first peak in incidence is often associated with participation in sport, such as football and racquet sports, whereas the second peak often occurs during normal daily activities, such as climbing stairs.^2^ However, all Achilles ruptures are associated with a pre-existing ‘tendinopathy’ which is attributed to failures in the protective/regenerative functions which respond to repeated microscopic injury.^3 4^

Traditionally, patients have been treated in plaster casts after rupture of the Achilles; with the cast immobilising the foot and ankle while the tendon heals. However, there are potential problems with this approach. First, there is the immediate impact on mobility for a period of around 8 weeks. Second, there are the complications and risks associated with prolonged immobilisation: muscle atrophy, deep vein thrombosis and joint stiffness.^5 6^ Finally, there are the potential long-term consequences which include prolonged gait abnormalities, persistent calf muscle weakness and an inability to return to previous activity levels.^7^ Functional bracing, involving immediate, protected weight-bearing in an orthotic, was designed to address these issues.

In patients having a surgical repair, seven RCTs^1 8–13^ were conducted, directly comparing plaster casts with early movement and/or weight-bearing in a ‘functional brace’. The results favour functional bracing in terms of re-rupture rate, functional outcome and quality of life measures. Therefore, in the first guideline (2009) produced on this topic, the American Academy of Orthopaedic Surgeons recommended functional bracing for patients having surgical repair of their tendon.^14^

We supplemented the 2004 Cochrane review^15^ with an updated literature search and found that in total only two studies^16 17^ been performed comparing the use of functional bracing with plaster casts for patients managed non-operatively following rupture of the Achilles tendon. Both studies suggested potential benefits from bracing. However, the data from the studies should be interpreted with caution owing to small patient numbers (90 in total), patients having received different functional bracing regimes and minimal reporting of outcomes. The gap in the evidence was recognised in the recent American Academy of Orthopaedic Surgeons, guideline 2009^14^ which concluded that ‘For patients treated non-operatively, we are unable to recommend for or against the use of immediate functional bracing for patients with acute Achilles tendon rupture’. Does functional bracing provide improved function and quality of life if the tendon is not surgically repaired? Or, in the context of a tendon that has not been stitched together, does a plaster cast provide greater protection and therefore improved healing? Does functional bracing facilitate faster return to work and is this cost effective? Or, is the tendon more vulnerable to re-rupture in a brace with the subsequent risk and cost of reconstructive surgery?

### Good clinical practice

The trial will be carried out in accordance with the Medical Research Council Good Clinical Practice and applicable UK legislation using the following protocol.

### Consort

The trial will be reported in line with the CONSORT statement using the non-pharmacological treatment interventions extension.

### Trial design

#### Aim

The aim of this project is to improve functional outcome by determining the best rehabilitation strategy for non-operatively managed patients with a rupture of the Achilles tendon.

#### Objectives

The primary objective is: to quantify and draw inferences on observed differences in Achilles Tendon Total Rupture Score (ATRS) between the trial treatment groups at 9 months after injury.

The secondary objectives are:

1. To quantify and draw inferences on observed differences in ATRS between the trial treatment groups at 8 weeks and 3 and 6 months after the injury,
2. To identify any differences in health-related quality of life between the trial treatment groups in the first 9 months after the injury,
3. To determine the complication rate between the trial treatment groups in the first 9 months after the injury,
4. To investigate, using appropriate statistical and economic analytical methods, the resource use, costs and comparative cost-effectiveness between the trial treatment groups.

### Outcome measures

The primary outcome measure for this study is the ***ATRS*.** 18 The ATRS is a validated questionnaire which is self-reported. It consists of 10 items assessing symptoms and physical activity specifically related to the Achilles tendon. It measures: strength, fatigue, stiffness, pain, activities of daily living, walking on uneven surfaces, walking upstairs or uphill, running, jumping and physical labour. This data will be collected at baseline, 8 weeks and 3, 6 and 9 months postrandomisation. The ATRS reaches a plateau between 6 and 9 months after rupture.^19^ The validity and reliability of this outcome measure has been previously published.^18 20^

The secondary outcome measures in this trial are:

Euroqol-5D: The EQ-5D-5L is a validated, generic health-related quality of life measure consisting of 5 dimensions each with a 5-level answer possibility. The EQ-5D can be used to report health-related quality of life in each of the five dimensions, and each combination of answers can be converted into a health utility score.^21^ It has good test–retest reliability, is simple for patients to use and gives a single preference-based index value for health status that can be used for broader cost-effectiveness comparative purposes. This data will be collected at baseline, 8 weeks and 3, 6 and 9 months postrandomisation.

*Complications*: All complications will be recorded, from the medical records at the 8-week review and self-reported by the patient thereafter. This data will be collected at 8 weeks and 3, 6 and 9 months postrandomisation.

### Sample size

The primary outcome for this study is the ATRS^18^. This is a 10-question, patient reported, outcome measure designed for patients with an Achilles tendon rupture. The individual items are converted to a 100-point scale where ‘0’ represents complete disability and ‘100’ is normal function. We have chosen a minimum clinically important difference (MCID) for the ATRS of 8 points.^20^ At an individual patient level, a difference of 8 points represents the ability to walk upstairs or run with ‘some difficulty’ versus with ‘great difficulty’. At a population level, 8 points represents the difference between a ‘healthy patient’ and a ‘patient with a minor disability’.^20^

The SD of the ATRS 9 months after injury in previous work was 20 points^19^; with an approximately normal distribution.

Based on this current knowledge, 264 patients represent the most likely scenario for 90% power to detect the selected MCID; representing a standardised effect size of 0.4.

Allowing a margin of 20% loss of primary outcome data, we propose to recruit a minimum of 330 patients in total. The 20% loss of data will include patients who cross-over between interventions and those who are lost to follow-up. Therefore, a minimum of 165 patients randomised to each group will provide 90% power to detect a difference of 8 points in ATRS at 9 months at the 5% level.

### Methodology

#### Screening

All adult patients presenting within 14 days of a primary (first-time) rupture of the Achilles tendon will be screened by the clinical care team in a minimum of 20 National Health Service (NHS) trust in the UK. The patient, in conjunction with their surgeon, will decide if they have surgery or not. If they decide not to have surgery (non-operative treatment), they will be eligible to take part in the trial and will be referred to the research team.

Screening logs will be collected throughout the trial to assess the main reasons for patient exclusion as well as number of patients unwilling to take part.

#### Eligibility

Patients will be considered for participation in this study if:

They are aged 16 years or older.

They have a primary rupture of the Achilles tendon.

They have decided to have non-operative treatment.

Patients will be excluded from participation in this study if they:

Present to the treating hospital more than 14 days after the injury,

There is evidence that the patient would be unable to adhere to trial procedures or complete questionnaires; for example, a history of permanent cognitive impairment.

The first exclusion criterion relates to patients with ‘late presentation’, which is not uncommon after this injury. Patients who present late may have problems with chronic tendon lengthening irrespective of treatment and are frequently offered surgical intervention. The ‘14 days’ has been widely used to define ‘acute’ rupture, including in our own pilot work.

The second criterion reflects the fact that much of the outcome data is collected by postal or electronic questionnaire, when help and support will not be available. Also, the primary outcome measure is not validated for proxy reporting.

If a patient taking part in the study were to sustain a contralateral rupture during the trial period, the second rupture would not be included in the study because the result of this intervention would not be independent from the first intervention. However, the patient would remain in the trial, with both previous and future data related to the initial rupture included in the final analysis.

#### Consent

Informed consent from the patient will be obtained by an appropriately trained and delegated member of the research team. Patients will be provided with verbal and written information about the study. Patients will be asked to consent to long-term follow-up and data linkage to routine NHS datasets.

#### Randomisation

The 1:1 allocation to either functional bracing or plaster cast will be generated by the trial statistician and concealed using a secure, centralised, computer-generated allocation sequence and web-based randomisation service. The Research Associate will inform the treating clinical team of the allocated treatment.

*Stratification by centre* will help to ensure that any effect related to the centre itself will be equally distributed in the trial arms. The catchment area will be similar for all of the hospitals; each hospital is a trauma unit dealing with these injuries on a daily basis. While unlikely, it is possible that the clinicians at one centre may be more expert in one or other treatment than those at another centre. Therefore, all of the recruiting hospitals have been/will be chosen on the basis that both techniques are currently routinely available at the centre, that is, the clinical staff are already familiar with both plaster casts and functional bracing.

#### Postrandomisation withdrawals

Participants may decline to continue to take part in the trial at any time without prejudice. A decision to decline consent or withdraw will not affect the standard of care the patient receives.

Participants have two options for withdrawal:

1. Participants may withdraw from completing any further questionnaires but allow the trial team to still view and record anonymously any relevant hospital data that is recorded as part of normal standard of care.
2. Participants can withdrawal wholly from the study, and only data obtained up until the point of withdrawal will be included in the final analysis of the study, thereafter no further data will be collected for that participant.

Withdrawn participants will not be replaced as the sample size calculation has allowed for loss to follow-up.

#### Blinding

As the type of rehabilitation used is clearly visible, the patients cannot be blind to their treatment. In addition, the treating clinician will also not be blind to the treatment but will take no part in the posttreatment assessment of the patients. The outcome data will be collected and entered onto the trial central database via questionnaire, by a research assistant/data clerk in the trial central office to reduce the risk of assessment bias.

### Trial treatments

Following randomisation, one group of patients will receive a plaster cast, and one group will receive a functional brace. All of the hospitals involved in this trial currently use both plaster casts and functional bracing for patients with both soft tissue injuries (tendon and ligament) and fractures, so all clinicians/units are familiar with both interventions.

Although the principles of application of both plaster casts and functional bracing are inherent in the technique, there are different types of plaster cast material and functional brace design. Each patient will undergo the allocated intervention according to the protocol below, but the details of the plaster and brace will be left to the discretion of the treating clinician, as per their usual practice. This will ensure that the results can be generalised across the NHS.

#### Plaster cast

The initial plaster cast will be applied in the ‘gravity equinus’ position, that is, the position that the foot naturally adopts when unsupported. In this position, with the toes pointing down towards the floor, the ends of the ruptured tendon are roughly approximated. Some units may use ultrasound to assess the approximation of the tendon ends, but this is not routine^22^ and so will be left to the discretion of the treating clinician. The patient may mobilise with crutches immediately using their toes for balance (toe-touch), but patients are not able to bear weight on the injured hindfoot. Over the first 8 weeks, as the tendon heals, the position of the plaster cast is changed until the foot achieves ‘plantargrade’, that is, the foot is flat to the floor. At this point, the patient may start to bear weight in the plaster cast. The number of changes of plaster cast and the time to weight-bearing will be left to the discretion of the treating clinician, as per their usual practice. The cast will be removed at 8 weeks.

The plaster cast provides maximum protection for the healing tendon, specifically it restricts the upward (dorsiflexion) movement of the ankle which may stretch the healing tendon, but it does not allow the patient to bear weight on the foot immediately.

#### Functional bracing

A rigid brace will be used in the trial, as opposed to a flexible brace.^23^ Initially, two 1 cm heel solid wedges (or equivalent) will be inserted into the brace to replicate the ‘gravity equinus’ position of the foot.^24^ The patient may mobilise with immediate full weight-bearing within the functional brace. The number of wedges/foot position will then be reduced until the patient reaches ‘plantigrade’. Again, the timing of the removal of wedges/change in foot position will be left to the discretion of the treating clinician, as per their usual practice. The brace will be removed at 8 weeks.

The functional brace does not provide the same restriction of movement as the cast but does allow the patient to bear weight on the foot immediately.

#### Rehabilitation

When the cast/brace is removed after 8 weeks, we will provide all patients (both groups) with the same standardised, written physiotherapy advice detailing the exercises they need to perform for rehabilitation following their injury. This will be based on a published systematic review of current rehabilitation protocols.^24^ All of the patients in both groups will be advised to move their toes, ankle and knee joints fully within the limits of their comfort, and walking will be encouraged. In this pragmatic trial, any other rehabilitation input beyond the written physiotherapy advice (including a formal referral to physiotherapy) will be left to the discretion of the treating clinicians. However, a record of any rehabilitation input (type and number of additional appointments) as well as other investigations/interventions will be requested as part of the 8-week, 3-month, 6-month and 9-month follow-up questionnaires.

### Adverse event management

Adverse events will be listed on the appropriate Case Report Form for routine return to the ‘UK STAR’ central office. Serious adverse events (SAEs) will be entered onto the SAE reporting form and reported to the central study team. However, some adverse events are foreseeable as part of the proposed treatment and will not be reported on an SAE reporting form but recorded on a complications form. These events include: re-rupture, blood clots/emboli, pressure areas/hindfoot pain, falls and neurological symptoms in the foot.

All participants experiencing SAEs will be followed-up as per protocol until the end of the trial. All unexpected SAEs that occur between date of consent and 9-month follow-up point will be reported to the sponsor and ethics committee.

### End of trial

The end of the trial will be defined as the collection of 9 months outcome data from the last participant.

### Statistical analysis

Results reporting will be in accordance with the CONSORT extension for non-pharmacological interventions.^25^ Primary analysis will be conducted on an intention-to-treat basis to compare the treatment arms in terms of the ATRS at 9 months after surgery. Early functional status will also be assessed and reported at 8 weeks and 3 and 6 months. Baseline characteristics will be summarised with descriptive statistics, presented for both treatment arms and overall.

We do not expect age or gender to influence the treatment effects, based on the results of our pilot work.^20^ However, the main findings of the trial will be reported as the difference on the ATRS between treatment arms, estimated with a linear mixed effects (LME) regression model, including outcome information from all follow-up points and adjusting for these and other potentially important prognostic variables. As individual clinicians will treat only a small number of patients, important clinician-specific effects are not expected; but, recruiting centre will be included in the model as a random effect factor to adjust for potential cluster differences. Estimates of treatment effects will be presented with 95% CIs. All statistical tests will be two-sided and considered to provide strong enough evidence to support differences between treatments if p values are smaller than 0.05 (5% significance level). Where severe departure from normal distribution is identified, data transformation will be endeavoured. If normality cannot be achieved, comparisons will be done using non-parametric tests without adjustment. Secondary continuous outcomes will be analysed using the same methods applied to the primary outcome.

All available data from both treatment arms will be used in the analysis. Although high amounts of missing data are not expected, its nature and pattern will be carefully considered. LME regression models have the advantage of producing robust estimates when data is missing at random. Nevertheless, missing data multiple imputation will be implemented if judged appropriate. In this case, sensitivity analysis will be conducted with the resulting imputed datasets. Reasons for ineligibility, withdrawal, non-compliance or other protocol violations will be described, and any patterns summarised. A detailed statistical analysis plan will be agreed early in the trial, and any subsequent amendments will be clearly stated and justified in the final study report. Interim analysis of the outcomes is not planned and will only be undertaken where directed by the independent data monitoring committee. Statistical analysis will be performed using validated statistical packages such as Stata (v14.2 or higher, www.stata.com) or R (www.r-project.org/).

### Health economic analysis

A prospective economic evaluation, conducted from an NHS and personal social services perspective, will be integrated into the trial design. The economic evaluation will estimate the difference in the cost of resource inputs used by participants in the two arms of the trial, allowing comparisons to be made between the two non-surgical treatment options (plaster cast vs functional bracing) for patients with a primary (first-time) rupture of the Achilles tendon and enabling costs and consequences to be compared. The economic assessment method will, as far as possible, adhere to the recommendations of the National Institute for Health and Care Excellence (NICE) Reference Case.^26^

Primary research methods will be followed to estimate the costs of the treatment options, including supplementary interventions (eg, surgery) and rehabilitation inputs. Broader resource utilisation will be captured through to principal sources: (1) routine health service data collection systems and (2) patient questionnaires administered at baseline, 8 weeks and 3, 6 and 9 months postrandomisation. Unit costs for health and social care resources will largely be derived from local and national sources and estimated in line with best practice. Primary research using established accounting methods may also be required to estimate unit costs. Costs will be standardised to current prices where possible. Health-related quality of life will be measured at baseline preinjury, at the time of consent, 8 weeks and 3, 6 and 9 months postrandomisation using the EuroQol EQ-5D-5L measure; responses will be used to generate quality-adjusted life-years (QALYs). Utility weights will be estimated using recommended algorithms until a national tariff set for the EQ-5D-5L is available.^21^ We will in the first instance use self-reports of the EQ-5D measure. Where these data are not available, we will estimate health utilities at each time point using mapping equations between the ATRS and EQ-5D health outcomes on the basis of existing datasets held by the trial team.

Multiple imputation methods will be used to impute missing data and avoid biases associated with complete case analysis. The results of the economic evaluation will be expressed in terms of incremental cost per QALY gained. We shall use non-parametric bootstrap estimation to derive 95% CIs for mean cost differences between the trial groups and to calculate 95% CIs for incremental cost--effectiveness ratios. A series of sensitivity analyses will be undertaken to explore the implications of uncertainty on the incremental cost-effectiveness ratios and to consider the broader issue of the generalisability of the study results. One such sensitivity analysis will involve adopting a societal perspective for the economic evaluation, which will incorporate direct costs to trial participants, informal care provided by family and friends and productivity losses. In the baseline analysis, and for each sensitivity analysis, cost-effectiveness acceptability curves will be constructed using the net-benefits approach. Heterogeneity in the trial population will be explored by formulating net-benefit values for trial participants from the observed costs and effects and then constructing a regression model with an intervention variable and covariates such as age and sex. The magnitude and significance of the coefficients on the interactions between the covariates and the intervention variable will provide estimates of cost-effectiveness of the treatment options by participant subgroup. More extensive economic modelling using decision-analytic methods will extend the target population, time horizon and decision context, drawing on best available information from the literature together with stakeholder consultations to supplement the trial data. Parameter uncertainty in the decision-analytic model will be explored using probabilistic sensitivity analysis. Longer-term costs and consequences will be discounted to present values using discount rates recommended for health technology appraisal in the UK.

### Trial oversight

The day-to-day management of the trial will be the responsibility of the Clinical Trial Manager, based at Nuffield Department of Orthopaedics, Rheumatology and Musculoskeletal Sciences and supported by the Oxford Clinical Trials Research Unit staff. This will be overseen by the Trial Management Group, who will meet monthly to assess progress. It will also be the responsibility of the Clinical Trial Manager to undertake training of the research associates at each of the trial centres. The Trial Statistician and Health Economist will be closely involved in setting up data capture systems, design of databases and clinical reporting forms.

A Trial Steering Committee (TSC) and a Data and Safety Monitoring Committee (DSMC) will be set up. The study DSMC will adopt a DAMOCLES (DAta MOnitoring Committees: Lessons, Ethics, Statistics) charter which defines its terms of reference and operation in relation to oversight of the trial. They will not be asked to perform any formal interim analyses of effectiveness. They will, however, see copies of data accrued to date or summaries of that data by treatment group, and they will assess the screening algorithm against the eligibility criteria. They will also consider emerging evidence from other related trials or research-related and review-related SAEs that have been reported. They may advise the chair of the TSC at any time if, in their view, the trial should be stopped for ethical reasons, including concerns about participant safety. DSMC meetings will be held at least annually during the recruitment phase of the study.

### Quality control

The study may be monitored or audited in accordance with the current approved protocol, relevant regulations and standard operating procedures by the host organisation, sponsor or appropriate regulatory authorities. A monitoring plan will be developed according to the OCTRU standard operating procedures which involve a risk assessment. The monitoring activities are based on the outcome of the risk assessment and may involve central monitoring and site monitoring.

### Ethics and dissemination

The National Research Ethic Committee approved this study on 18 March 2016 (16/SC/0109).

The study monograph for the National Institute for Health Research Health Technology Assessment will be prepared by the trial management team within 3 months of completion of the trial. A manuscript for a high impact peer-reviewed journal will be prepared simultaneously, which will allow for the results to be disseminated across the orthopaedic and rehabilitation communities, the wider medical community, NICE and policy-makers. Authorship will be determined in accordance with the International Committee of Medical Journal Editors guidelines, and other contributors will be acknowledged. The results of this trial will substantially inform clinical practice on the clinical and cost-effectiveness of the treatment of this injury. The results of this project will be disseminated to patients via patient-specific newsletters and through local mechanisms at all participating centres.

## Supplementary Material

Reviewer comments
