# Supplementary material for: Cast versus functional brace in the rehabilitation of patients treated non-operatively for a rupture of the Achilles tendon: protocol for the UK study of tendo achilles rehabilitation (UK STAR) multi-centre randomised trial
Source: BMJ Open. 2017 Oct 24;7(10):e019628. doi: 10.1136/bmjopen-2017-019628 (PMC5665283; doi:10.1136/bmjopen-2017-019628)
Supplement: Reviewer comments [file bmjopen-2017-019628.reviewer_comments.pdf]

PEER REVIEW HISTORY

BMJ Open publishes all reviews undertaken for accepted manuscripts. Reviewers are asked to complete a checklist review form (**http://bmjopen.bmj.com/site/about/resources/checklist.pdf**) and are provided with free text boxes to elaborate on their assessment. These free text comments are reproduced below.

ARTICLE DETAILS

**TITLE (PROVISIONAL)**	Cast versus functional brace in the rehabilitation of patients treated non-operatively for a rupture of the Achilles tendon: protocol for the UK Study of Tendo Achilles Rehabilitation (UK STAR) multi-centre randomised trial
**AUTHORS**	Achten, Juul; Parsons, Nicholas; Kearney, R.S.; Maia Schlüssel, Michael; Liew, Anna; Dutton, Susan; Petrou, Stavros; Ollivere, Benjamin; Lamb, Sarah; Costa, Matthew

This article was not externally reviewed at BMJ Open. Protocols that have been independently assessed prior to submission to BMJ Open are usually fast-tracked to publication on the grounds that further substantial changes will not be possible. This independent assessment will usually be external, independent review for both a substantial grant award from a non-commercial or government funder and ethics approval.
